# Accelerometer-based analyses of animal sleep patterns

**DOI:** 10.7554/eLife.77349

**Published:** 2022-03-08

**Authors:** Yuuki Y Watanabe, Christian Rutz

**Affiliations:** 1 National Institute of Polar Research Tokyo Japan; 2 Department of Polar Science, The Graduate University for Advanced Studies, SOKENDAI Tokyo Japan; 3 Centre for Biological Diversity, University of St Andrews St Andrews United Kingdom

**Keywords:** sleep, homeostasis, olive baboon, predation risk, social behavior, biotelemetry, Other

## Abstract

Body-motion sensors can be used to study non-invasively how animals sleep in the wild, opening up exciting opportunities for comparative analyses across species.

**Related research article** Loftus JC, Harel R, Núñez CL, Crofoot MC. 2022. Ecological and social pressures interfere with homeostatic sleep regulation in the wild. *eLife* **11**:e73695. doi: 10.7554/eLife.73695.

Presumably, it is not just humans who enjoy a good night’s sleep. But we actually know surprisingly little about the sleep habits of other animals, not least because of significant methodological challenges. Recent research suggests that small sensors that record body motion – similar to those used in wearable fitness devices – could become a game changer in animal sleep research.

Sleep in humans and other animals is typically studied under controlled conditions using an electroencephalogram (EEG), which measures electrical activity in the brain. The miniaturization of animal-borne EEG loggers has recently made it possible to record sleep in wild animals, in its full ecological context. Amongst other things, these ‘neuro-loggers’ revealed that sloths sleep less than previously thought and confirmed that some seabirds nap on the wing (Rattenborg et al., 2008; Rattenborg et al., 2016). But the invasive nature of the method, which – unlike routine human EEG applications – requires surgical implantation of electrodes, is a concern and limits the loggers’ wider applicability.

A promising non-invasive alternative is to record body motion using animal-borne accelerometers to pinpoint periods of sustained inactivity. This is the same approach smart watches and fitness wristbands employ, which tell their human users how many steps they have walked, praise their fat-burning efforts, or indeed, warn them if they have not slept enough (Williams et al., 2021). Body motion is of course only a proxy measure, and there is a risk that wakeful resting periods are misidentified as ‘sleep’. Nevertheless, this easy-to-use technology has enormous potential for studying sleep, as demonstrated by studies on both human and non-human subjects (Jones et al., 2019).

Now, in eLife, Carter Loftus, Roi Harel, Chase Núñez and Margaret Crofoot report on the use of animal-borne accelerometers to map the sleep patterns of a free-ranging group of baboons in Kenya (Loftus et al., 2022). Collars, which also housed GPS loggers for high-resolution movement tracking, were fitted to 26 adults, yielding data for more than 500 nights. Back in the office, the acceleration readings were analysed using a sleep classification algorithm that had been developed for human applications, and results were validated using field-recorded infrared video footage (Figure 1).

**Figure 1.**
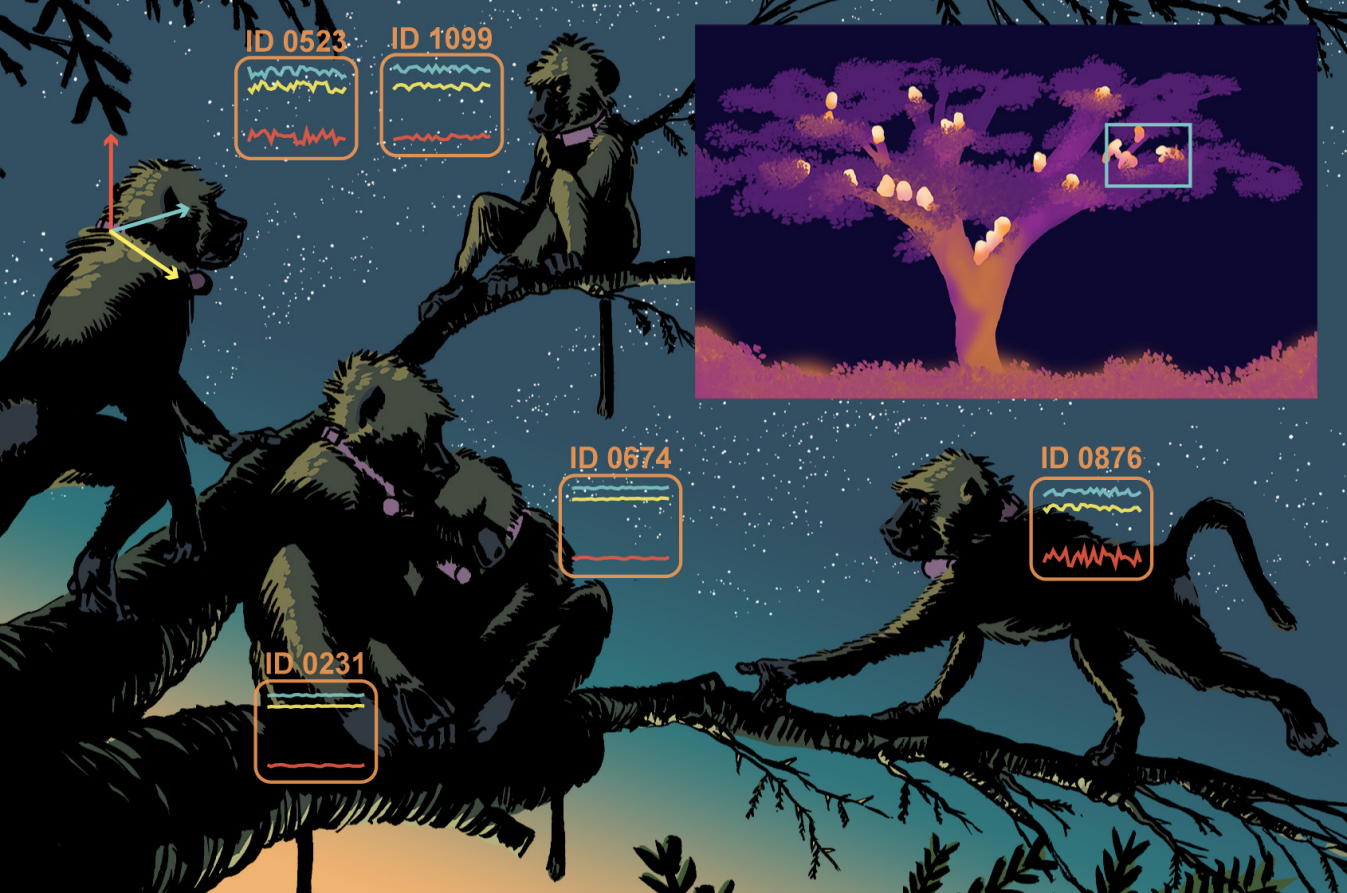
Investigating sleep patterns in a group of baboons. Collar-mounted accelerometers record fine-scale body movements along three axes – *x* (yellow), *y* (blue), and *z* (red). An algorithm later identifies periods of sustained inactivity and classifies them as 'sleep' (animals: ID 0231, ID 0674). Infrared video recordings (inset) of tagged baboons at their sleep site are used to validate these classifications. Distinguishing sleep (ID 0231, ID 0674) from resting wakefulness (ID 1099) using this non-invasive method remains a challenge.

Image credit: Mike Costelloe, for the Max Planck Institute of Animal Behavior (CC-BY-NC-ND 4.0).

Using this accelerometer-based approach, the researchers obtained fascinating glimpses of how ecological and social factors affect the timing and duration of sleep. For example, the tagged baboons slept less when spending the night in unfamiliar trees, or in proximity to a larger number of groupmates. In contrast, the degree of physical exhaustion following daytime travel, and the amount of time slept the night before, had only limited effects on their sleep behaviour. Perhaps surprisingly, wakeful periods were largely synchronized within the group, instead of ‘sentinels’ taking turns watching out for danger. While such synchronization may have social benefits, it certainly also has its risks – as illustrated by an (unsuccessful) nocturnal leopard attack that occurred during the observation period.

Loftus et al. – who are based at the University of California at Davis, the Max Planck Institute of Animal Behavior, the University of Konstanz and the Mpala Research Centre – provide a compelling demonstration that it is possible to record the sleep patterns of wild animals over extended periods of time, using routine animal-borne technology. Accelerometers can be attached easily and safely to a wide range of species, without the need for surgery. In fact, over the past two decades, these loggers have become an indispensable component of the research toolkit available for studying wild animals. Amongst other applications, they are being used to chart activity profiles, to estimate energy expenditure, and to detect difficult-to-observe behaviours (Yoda et al., 2001; Wilson et al., 2006; Watanabe and Takahashi, 2013). Yet, despite the success of a first wave of pioneering studies, the potential of accelerometers as ‘sleep detectors’ remains to be fully exploited (e.g., Miller et al., 2008; Samson et al., 2018; for additional references, see Loftus et al., 2022).

There is an exciting opportunity to advance animal sleep research. Every year, biologists are deploying accelerometers on thousands of wild animals. There will no doubt be many existing datasets that can be analysed retrospectively, to search for the characteristic signatures of sleep. Furthermore, when planning new projects, researchers may wish to consider keeping their loggers switched on around the clock, rather than pausing data collection at night to preserve battery life. While accelerometer-based sleep classification does not work for all taxa, it can massively boost phylogenetic coverage. Studying different species in their natural habitats will help to explore how sleep patterns are shaped by physiological, environmental, social and other factors.

A comparative research programme has the potential to address long-standing questions about animal sleep. Moreover, such work could make innovative contributions to conservation science by helping us understand how animals manage their sleep requirements in the face of humanity’s relentless hustle and bustle (Rutz et al., 2020).
